# Some Thoughts and Experiments upon Erosion

**Published:** 1886-04

**Authors:** Edgar D. Swain

**Affiliations:** Chicago, Ill.


### SOME THOUGHTS AND EXPERIMENTS UPON EROSION.

BY EDGAR D. SWAIN, D. D S., CHICAGO, ILL.

In the literature of our profession we find the terms Denudation, Abrasion, Mechanical Abrasion, Chemical Abrasion, Spontaneous Abrasion and Erosion, employed to convey to the mind of readers two conditions, which, it seems to me, could better be expressed by the two terms of Mechanical Abrasion and Erosion. Mechanical Abrasion conveys to the mind that condition with which all practitioners are familiar, namely, a gradual loss of tooth substance, produced by the attrition of the teeth upon one another, or of foreign bodies upon them. Erosion signifies the action of a corrosive substance, or the destruction of a part by a chemical agent, and applies equally well if the theory of destruction by micrococci be admitted.

Mechanical abrasion is of so frequent occurrence, and the methods used by our profession to arrest its progress are so well understood, that I pass it with the simple recognition that it is successfully arrested by the methods adopted by us.

Erosion, we will assume, covers all those conditions with which we are no less familiar, but the causes of which are less patent. Under this head we find the gradual wasting of an individual tooth, or of many, the wasting of the anterior teeth upon their incisive edges, or the cutting of grooves across their labial surfaces, and sometimes regular and irregular patches upon their labial and proximal surfaces.

Under the first condition the anterior teeth generally present an elliptic-shaped space between the upper and lower incisors, when the back teeth are closed. In these cases the destructive force can not be attributed to mechanical forces, because they can no longer be brought into contact with one another, and this condition can only be accounted for either by the theory of insufficient nutrition, the subsequent death of animal tissue and consequent falling to pieces of the mineral portion, or its destruction by some corrosive substance. A theory has been advanced to the effect that glands located in the end of the tongue become diseased, and secrete a substance sufficiently destructive to accomplish this result.

My own opinion is that we need no theory to better account for this kind of destruction than that of a deranged nutrition.

I have never, with teeth affected by this disease, been able to determine with the microscope that the terminal ends of the tubuli were filled with calcific material, or a deposit of any kind. Indeed, great difficulty is experienced in preparing specimens from such teeth for examination, because of the tendency to crumble, showing thereby a deficiency of the animal tissue. Such an attempt to resist the approach of destruction toward the pulp would not be consistent with a defective nutrition.

Teeth thus affected are never sensitive upon the eroded surface, and very seldom does the pulp become exposed, it receding and depositing secondary dentine, in amount about equal to the external waste in depth.

That feature of this disease which affects the labial surfaces of the anterior teeth, horizontally, about or just below the union of enamel and cementum, resulting in a groove or patch, with none of the characteristics of caries, no softening of the dentine, the entire groove presenting sharply defined edges and a highly polished surface, precludes even a possibility of mechanical causes. Teeth affected in this manner do not seem to be subject to caries, but usually have the appearance of a perfect development, clean, with a well-polished enamel. The etiology of this disease has been a stumbling block in the research of all our authors upon dental diseases.

Mr. John Tomes believes it to be of a strictly mechanical origin.

Garretson, a disease of predisposition, the result of impressions at the time of enamel development, or that it is a result of electrolysis.

Fox looks to an abnormal condition of the secretions of the oral cavity and friction of the lips.

Hunter believes it a disease inherent in the tooth, and not dependent on circumstances in after life.

Harris attributes it to acidulated buccal mucus.

Taft advances no opinion.

Some have advanced the theory of a corrosive fluid secreted by the gingival margins of the gums, combined with a predisposition of the teeth to this disease, as the cause of destruction.

Leber and Magitot ascribe the destruction to leptothrix, which they found occupying the tubuli. Mr. Chas. Tomes believes that the solution of the question is to be disposed of upon the chemical theory, and argues that it is probable that mucus, by fermenting, affords an acid solvent.

Others have advanced the theory of absorption of the tooth substance. This is, of course, in direct antagonism to all theories of devitalization from a lack of nutrition, as enamel sufficiently deficient in vital force to resist the corroding influences would not have the necessary vital force to carry on the process of absorption.

Another class of writers have advanced the idea that the gingival margins of the gums secreted a fluid which acted upon the enamel, precisely as in the case of the absorption of the roots of temporary teeth.

This theory assumes that the process of absorption of deciduous teeth is carried on by the acid of a fluid solvent, mechanico-chemical (if I may use such a term) more than purely vital or physiological in its action, the reverse of building up.

I have said above that Garretson favored a theory of electrolysis, to which I again return.

On page 505 of his "Oral Surgery" he says: "The present conviction of the author is that the true explanation is just now for the first time enunciated in the electro-chemical experiments made by Mr. Kincely Bridgman, and that in this direction will be found to lie not alone the cause, but the prophylaxis."

The experiments referred to were conducted by Mr. Bridgman for the purpose or sustaining an advanced theory regarding the cause and formation of dental caries. Mr. Garretson seems ready to adopt the results as accounting for the phenomena of erosion as well.

The experiments consisted in placing in cold dilute sulphuric acid rods of pure zinc, amalgamated with freshly distilled mercury, one-half the rod immersed in the fluid while the other half remained in contact with the atmosphere. I use his language to explain the results: "In a very short time bubbles of hydrogen made their appearance over the whole surface exposed to the acid, and in forty-eight hours the metal was found to have lost upwards of ten grains in weight. This loss, however, was by far the least important part of the result. The immersed portion of the metal had not been acted upon uniformly over its whole surface, but the action had been greatest at the surface of the liquid. At the same time the exposed portion had become covered with patches of crystalline sulphate of zinc, high and dry upon the projecting portion of the metal. Therefore, not only had chemical action been excited between the metal and the acid and the water decomposed, but there was the additional evidence that the metal itself had become polarized."

Another similar experiment, in which copper was used, varied in results only in the sulphate of that metal being deposited upon its exposed surface and corroding the wire half way through, at the line between the fluid and atmosphere.

Another experiment to prove that this action which arises between the metal and acid was due to polarization followed, which consisted in wholly immersing a similar piece of wire in the acidulated water, where it remained for months without imparting the slightest color to the fluid; but when by evaporation the wire became exposed to the atmosphere, chemical action again commenced.

Further, he says: "The atmosphere in its normal state being electro-positive, by a well known law of induction renders bodies opposed to it electro-negative. The exposed end of the copper is, therefore, thus rendered electro-negative, and the acid by the same rule being electro-negative also, the immersed end of the metal becomes electro-positive. It is an established rule that bodies to be electro-decomposed must first be rendered electro-positive, and also that bodies receiving an addition of matter must first be made electro negative."

"The appearance, however, of the crystallization upon what was at first the dry end of the metal, requires particular attention. It is one of the special effects of the electrolytic action that fluids pass to, and accumulate at, the negative pole. Obeying this law, the acid immediately begins to ascend and spread itself over the surface of the fluid, and, by capillary attraction, over the exposed end of metal, from which point it evaporates, having deposited its chemical product." There is very much more in explanation of the electro-chemical phenomena, but what I have quoted will suffice my purpose. Let us now apply the theory to the teeth, which take the place of the wire. That portion beneath the margins of the gums becomes the electro-positive, while the crown of the tooth is the electro-negative. The constant throwing off and decomposing of worn-out epithelial cells and other foreign matter, with the saliva, provides the necessary acid fluid.

As I could not learn that Mr. Bridgman had used teeth, instead of metals, in his experiments, I placed in a weak solution of sulphuric acid and water several teeth and a copper wire. The results fully determined the electro-chemical action upon both teeth and wire. Upon the wire a deposit of sulphate of copper, and upon the ends of the teeth (the crowns) exposed to the atmosphere, a deposit of sulphate of lime was found.

One of the teeth so used was prepared by cutting along its side a groove, which was filled with amalgam. I observed no difference between the chemical action upon this tooth and the others. Sufficient time was not consumed in these experiments to determine whether erosion could be artificially produced by this means.

It was observed, however, that as the solution was diluted from day to day, as it was during the last four days of the experiments, the deposits upon the teeth became more copious.

Mr. Bridgman, in drawing his conclusions, has failed to recognize a vital resistive force which must exist in teeth in the jaws, and also the fact that the fluids surrounding the teeth at the margins of the gums are, during waking hours, subject to constant change. Whatever the agent may be which produces the erosion, its action, we know, is very slow, and it may be possible that it would accomplish its destructive work during the hours of repose.

Some years ago I became convinced that erosion was not the result of mechanical causes. The worst case I ever saw, where the lower incisors were severed to, and in one tooth beyond, the pulp cavity, was in a mouth where a tooth brush had never been used. A neighbor dentist had a similar case about the same time. It was learned that both men were constant smokers of cigarettes, and when comparing notes we felt sure that we had discovered one of the causes of erosion. Two swallows do not, however, make a summer, nor do all the people whose teeth are affected with this disease smoke cigarettes; but we do find in these two cases conditions which could be favorably construed to the support of the chemical theory. The burning of paper, among other products, gives carbonic dioxide, which would naturally be a constant element in the mouth, and especially about the lower incisors. This, however, would account for a very insignificant number of the cases where erosion is met with, and consequently compel further investigation to account for the other cases.

A theory of caries advanced by a writer in one of our dental journals may be mentioned in this connection, namely, that destruction of the teeth by disease anterior to amalgam fillings is always greater than behind them; or, in other words, the presence of an amalgam filling in the first molar tooth has the effect to so change the condition of the nerve (or vital) forces, that teeth anterior to it become weaker, and are less able to resist disease, but as erosion (as well as caries) occurs where no amalgam has been used, we must drop such a theory as unlikely to give us any help in the direction sought.

So far, I am not personally convinced that any one of the theories advanced throws much light upon this subject. Still, they may be of benefit to those interested, and possibly help some other individual who desires to investigate the causes of this growing disease.

That it is not caries, and is, primarily, a disease of the enamel, I believe to be true.

It is my intention to continue experiments in this direction, and I shall report progress at some future time.

